# Genetic association of circulating C-reactive protein levels with idiopathic pulmonary fibrosis: a two-sample Mendelian randomization study

**DOI:** 10.1186/s12931-022-02309-x

**Published:** 2023-01-09

**Authors:** Kun Zhang, Anqi Li, Jiejun Zhou, Chaoguo Zhang, Mingwei Chen

**Affiliations:** Department of Respiratory and Critical Care Medicine, First Affiliated Hospital of Xi’an Jiaotong University, No. 277 Yanta West Road, Xi’an, 710061 Shaanxi People’s Republic of China

**Keywords:** IPF, CRP, Mendelian randomization, GWAS

## Abstract

**Background:**

Several observational studies have found that idiopathic pulmonary fibrosis (IPF) is often accompanied by elevated circulating C-reactive protein (CRP) levels. However, the causal relationship between them remains to be determined. Therefore, our study aimed to explore the causal effect of circulating CRP levels on IPF risk by the two-sample Mendelian randomization (MR) analysis.

**Methods:**

We analyzed the data from two genome-wide association studies (GWAS) of European ancestry, including circulating CRP levels (204,402 individuals) and IPF (1028 cases and 196,986 controls). We primarily used inverse variance weighted (IVW) to assess the causal effect of circulating CRP levels on IPF risk. MR-Egger regression and MR-PRESSO global test were used to determine pleiotropy. Heterogeneity was examined with Cochran's Q test. The leave-one-out analysis tested the robustness of the results.

**Results:**

We obtained 54 SNPs as instrumental variables (IVs) for circulating CRP levels, and these IVs had no significant horizontal pleiotropy, heterogeneity, or bias. MR analysis revealed a causal effect between elevated circulating CRP levels and increased risk of IPF (OR_IVW_ = 1.446, 95% CI 1.128–1.854, *P* = 0.004).

**Conclusions:**

The present study indicated that elevated circulating CRP levels could increase the risk of developing IPF in people of European ancestry.

**Supplementary Information:**

The online version contains supplementary material available at 10.1186/s12931-022-02309-x.

## Background

Idiopathic pulmonary fibrosis (IPF) is an aggressive, irreversible lung disease marked by scar formation caused by an atypical response to epithelial injury [1]. IPF has become a worldwide public health problem. An epidemiological study found that the incidence of IPF is increasing over time worldwide. The annual incidence in North American and European populations is 3–9/100000, while the incidence in East Asia and South America is lower than that [2]. Furthermore, the prognosis for IPF is poor, with a median survival of only 2.5–3.5 years from diagnosis [3, 4]. Thus, early identifying the underlying risk factors for IPF can help to prevent IPF.

IPF was initially considered an inflammatory disease [5]. Later, investigators found that inflammation is involved in different stages of IPF development due to the activation of the innate and adaptive immune systems [6–8]. Environmental influences and genetic risk factors leading to chronic inflammation may be associated with the development of IPF [9]. C-reactive protein (CRP) is often considered a marker of the inflammatory response in the acute phase. Previous studies have found increased CRP concentrations associated with the risk of cardiovascular disease, psoriatic arthritis, type 2 diabetes, and cancer [10–14]. And some recent studies have found that CRP is also an important marker of chronic inflammation and may have an etiological role in cancer [10]. Several retrospective studies on IPF indicated that elevated circulating CRP levels are significantly related to poor survival in IPF [15] and may predict mortality during acute exacerbations of IPF [16, 17]. Interestingly, a Mendelian randomization (MR) study found a negative association between CRP and the genetic risk of IPF [18]. In short, the following aspects were considered: (1) there are fewer studies on the causal relationship of circulating CRP levels on the risk of IPF prevalence; (2) some contradictory results have emerged from these studies; (3) observational studies are likely to be affected by potential confounders or reverse causality bias that prevents reliable conclusions from being drawn. Therefore, it is necessary to clarify further the causal effect of circulating CRP levels on IPF.

MR analysis is a new epidemiological approach that uses genetic variations as instrumental variables (IVs) to estimate the causal relationship between exposure and outcome [19, 20]. Given that genetic variants are randomly assigned at conception, usually independent of environmental risk factors, and precede disease onset, MR analysis can avoid the effects of reverse causality and unmeasured confounders [20].

In short, the present research intended to assess the causal effect of circulating CRP levels on the risk of developing IPF by a two-sample MR approach using the summary statistics from two large sample genome-wide association studies (GWAS) of European ancestry.

## Methods

### Data source

To identify genetic loci related to circulating CRP levels, we utilized data from a large-scale GWAS meta-analysis of 88 studies (including 204,402 individuals) [21]. This GWAS meta-analysis revealed 58 genome-wide significant genetic loci for circulating CRP levels, explaining up to 7.0% of the variance in circulating CRP levels [21]. We used GWAS analysis of IPF from FinnGen biobank (freeze 5) as outcome variables, including genotype data of 1028 IPF patients and 196,986 controls [22]. The populations in both of the above GWAS analyses were of European ancestry. And the summary data of both GWAS analyses can be downloaded from the open-access GWAS dataset at https://gwas.mrcieu.ac.uk/ (CRP GWAS ID: ieu-b-35; IPF GWAS ID: finn-b-IPF).

### IVs for circulating CRP levels

**Fig. 1:**
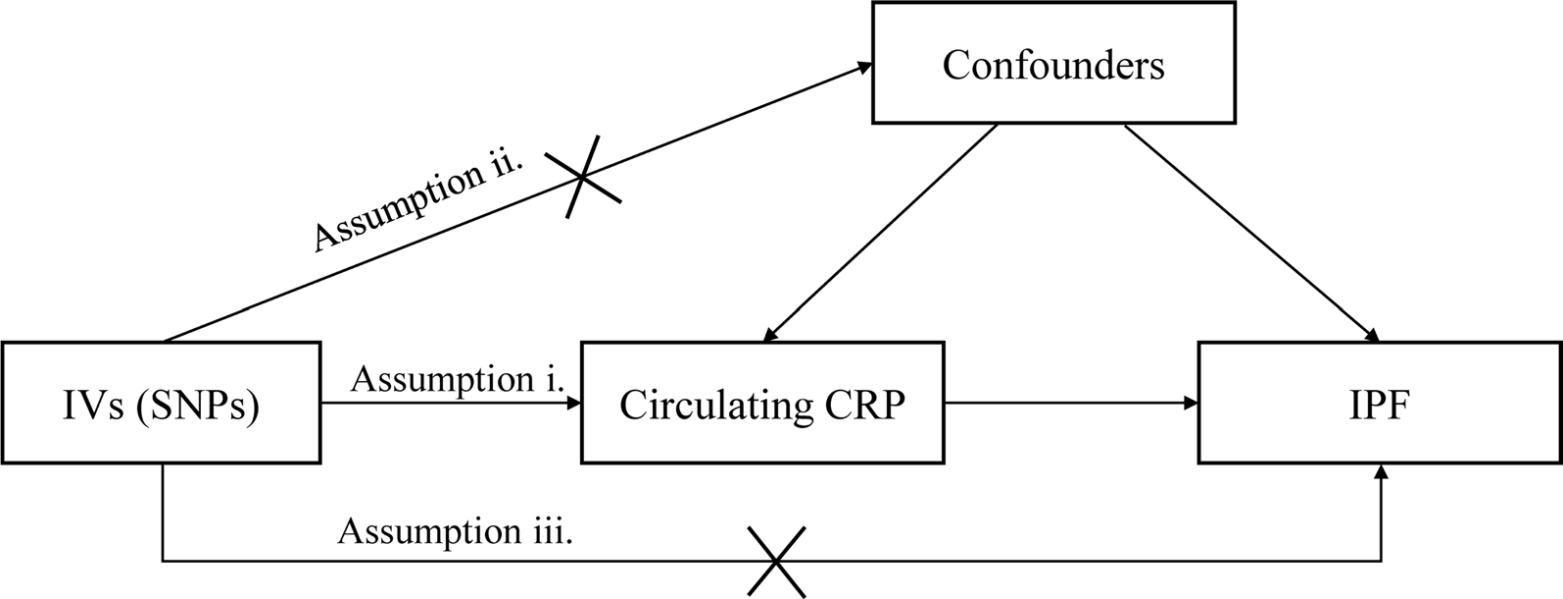
Three assumptions for IVs in MR analysis

To use genetic variation to assess the causal association between exposure (circulating CRP levels) and outcome (IPF), it must satisfy three critical assumptions for Ivs [20]: (i) IVs are related to circulating CRP at a genome-wide significant level; (ii) IVs must be independent of any confounders; (iii.) IVs affect IPF only through circulating CRP levels (Fig. 1). Due to the linkage disequilibrium structure in the genome, significant associations between genetic variants and traits were identified at a *P* = 5 × 10^–8^ threshold and r^2^ < 0.001 [23]. Then, we obtained 57 single nucleotide polymorphisms (SNPs) that were significantly related to circulating CRP levels using RStudio 4.1.1 and the package TwoSampleMR, which satisfied the first assumption (Additional file 1). Then, we extracted the data of 56 SNPs out of the above 57 SNPs from IPF GWAS, because rs644234 had no data in IPF GWAS (Additional file 2). In addition, we removed the following SNPs for being palindromic with intermediate allele frequencies: rs10778215, and rs11108056. Finally, we used 54 SNPs as IVs for circulating CRP levels in our study (Additional file 3).

### Statistical analysis

A two-sample MR analysis was utilized to examine the genetic relationship between circulating CRP levels and IPF risk. Inverse variance weighted (IVW) [24] was utilized as the major analytic approach, while MR-Egger [25], weighted median [26], weighted mode [27], and simple mode [28] were complementary methods. Then, we performed the sensitivity analyses and indirectly tested the second and third assumptions. Firstly, we tested the horizontal pleiotropy of IVs using the MR-PRESSO global test [29] and MR-Egger regression [25]. Secondly, Cochran's Q test was employed to determine heterogeneity among Ivs [30]. Additionally, we performed the Leave-one-out analysis to determine the undue influence of individual SNPs on the estimation of MR [31]. We performed MR analysis in RStudio 4.1.1 software utilizing the R package TwoSampleMR (version 0.5.6). *P* < 0.05 was considered significant.

## Results

**Fig. 2:**
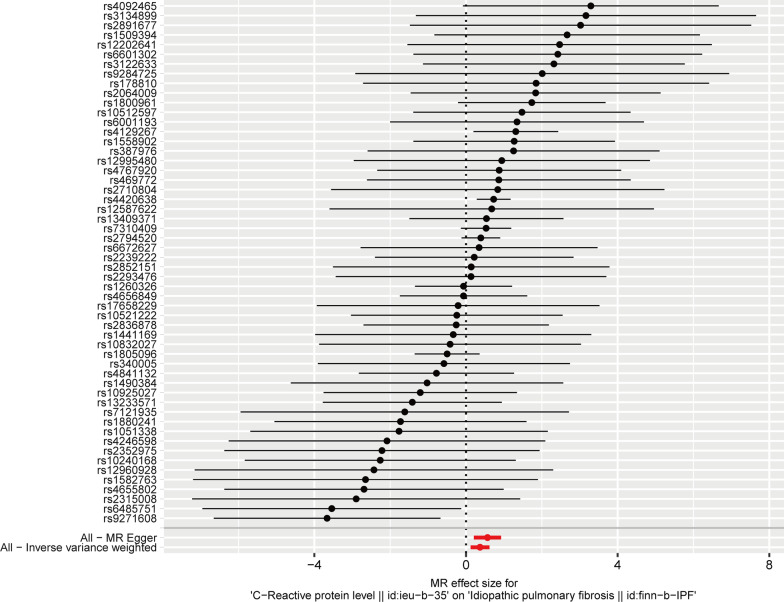
Forest plot for the causal effect of each SNP on IPF risk

**Fig. 3:**
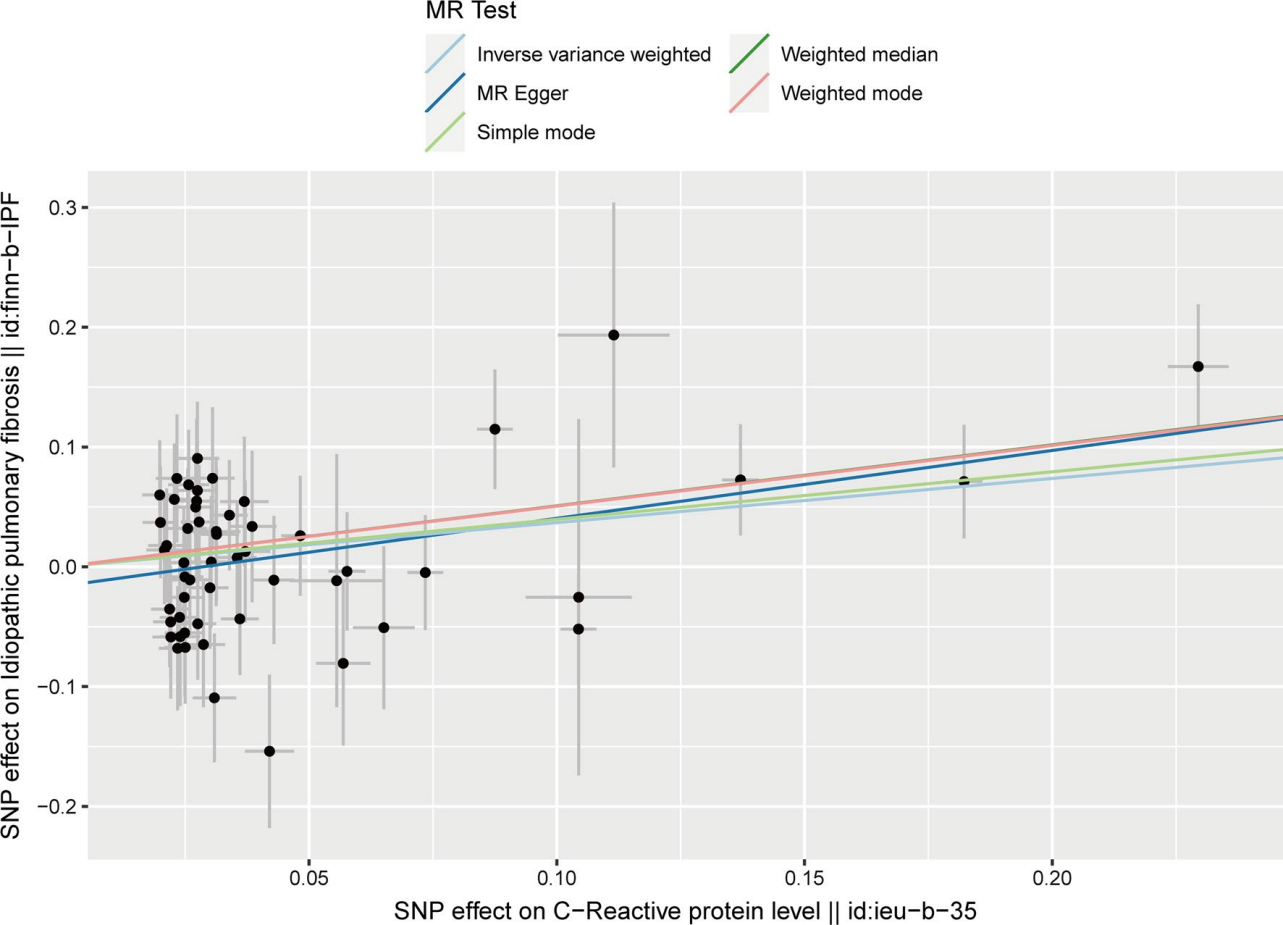
Scatter plot for the causal effect of circulating CRP levels on IPF risk

We obtained 54 SNPs as IVs to assess the genetic association of circulating CRP levels with IPF, and the causal effect of each SNP on IPF is shown in the forest plot (Fig. 2). Then, we performed MR analysis using these 54 SNPs, and the results of the IVW method showed a causal effect of the circulating CRP levels on the risk of IPF (OR_IVW_ = 1.446, 95% CI 1.128–1.854, *P* = 0.004) (Table 1). And MR-Egger (OR = 1.762, 95% CI 1.232–2.521, *P* = 0.003), weighted median (OR = 1.663, 95% CI 1.170–2.364, *P* = 0.005) and weighted mode ((OR = 1.660, 95% CI 1.250–2.203, *P* = 0.001) methods also yielded results consistent with the IVW method (Table 1). As shown in the scatter plot, the risk of developing IPF increases with the increasing circulating CRP levels (Fig. 3).

**Table 1:** Causal effect of circulating CRP levels on IPF

Exposure	Outcome	N(SNP)	Method	OR (95% CI)	*P*
Circulating CRP	IPF	54	MR Egger	1.762 (1.232–2.521)	0.003
Circulating CRP	IPF	54	Weighted median	1.663 (1.170–2.364)	0.005
Circulating CRP	IPF	54	Inverse variance weighted	1.446 (1.128–1.854)	0.004
Circulating CRP	IPF	54	Simple mode	1.487 (0.619–3.573)	0.379
Circulating CRP	IPF	54	Weighted mode	1.660 (1.250–2.203)	0.001

CRP: C-reactive protein; IPF: idiopathic pulmonary fibrosis; SNP: single-nucleotide polymorphism; OR: odds ratio; CI: confidence interval

**Fig. 4:**
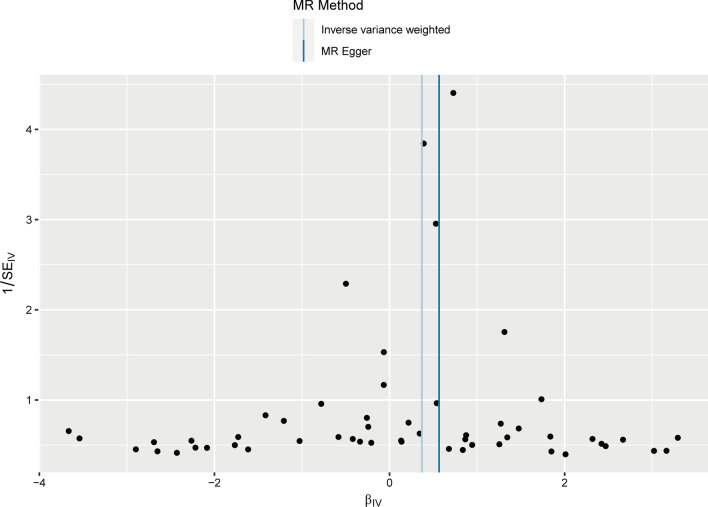
Funnel plot for the overall heterogeneity in the effect of circulating CRP levels on IPF risk

**Fig. 5:**
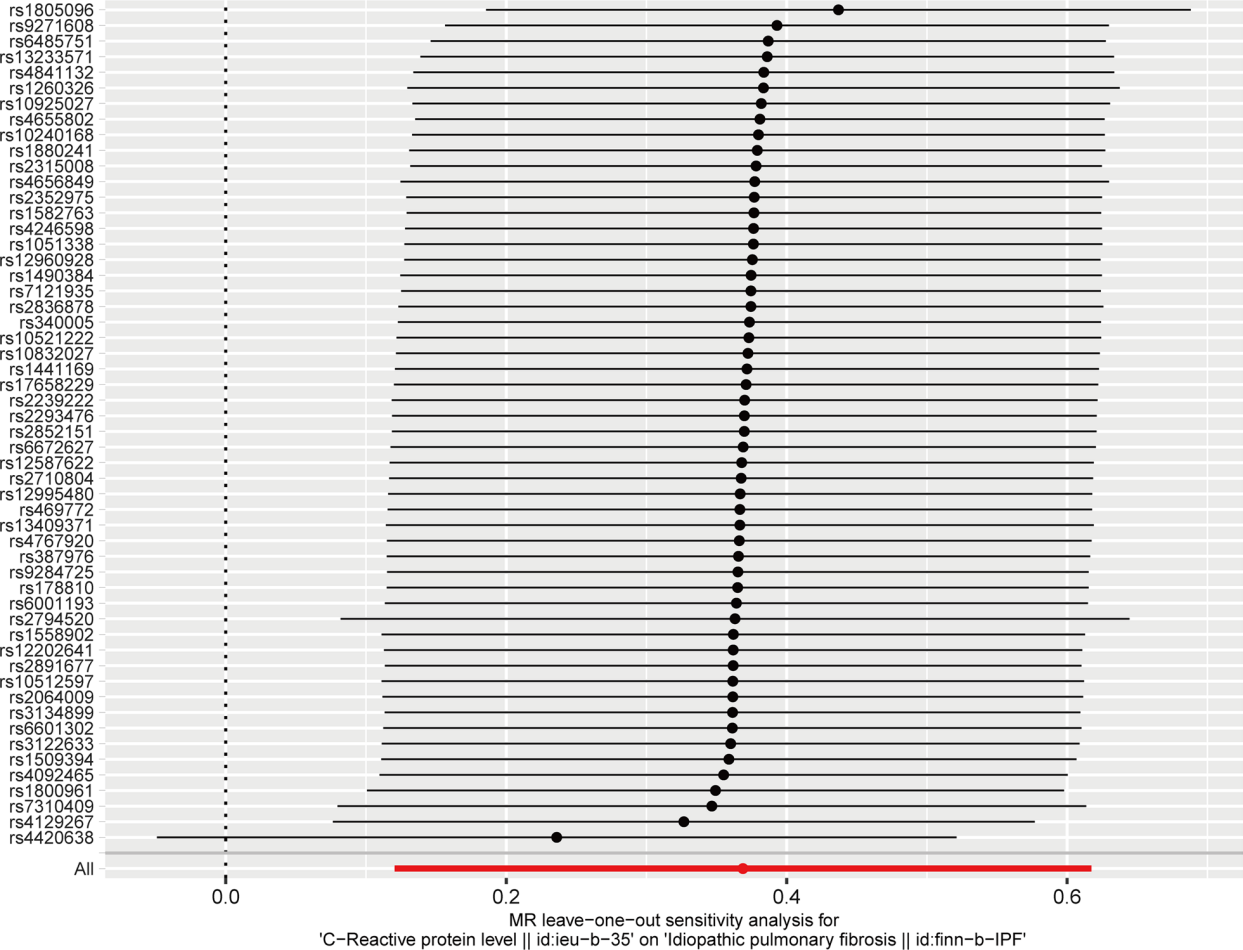
Leave-one-out analysis of the effect of circulating CRP levels on IPF

Subsequently, we performed sensitivity analyses to assess our results. Firstly, Cochran's Q test results suggested no heterogeneity among IVs (*P*_IVW_ = 0.204, *P*_MR Egger_ = 0.242, Table 2). The symmetry of the funnel plot also confirmed the absence of heterogeneity (Fig. 4). Secondly, no overall horizontal pleiotropy existed in all IVs, as shown by the results of the MR-PRESSO global test (*P* = 0.170, Table 2) and MR-Egger regression (*P* = 0.143, Table 2). This result suggests that IVs are unlikely to affect IPF risk through pathways other than circulating CRP levels. The leave-one-out sensitivity analysis by removing one SNP at a time showed stable results except for rs4420638 (Fig. 5).

**Table 2:** Pleiotropy and heterogeneity tests of MR

Test	Method	Effect size	*P*
Heterogeneity	Cochran's Q test	58.745 (Q_MR Egger_)	0.242
	Cochran's Q test	61.238 (Q_IVW_)	0.204
Pleiotropy	MR-Egger regression	− 0.016 (egger_intercept)	0.143
	MR-PRESSO global test	64.970 (RSSobs)	0.170

MR: Mendelian randomization; IVW: inverse variance weighted; RSS: residual sum of squares

## Discussion

Our study explored the causal effect of circulating CRP levels on the risk of IPF using a two-sample MR analysis. The results showed that elevated circulating CRP levels could lead to an increased risk of IPF. Moreover, sensitivity analysis suggested that our results were robust.

Our results provide one piece of evidence for the previously controversial conclusion. A proteomics study by Niu et al. identified that CRP might be a potential specific biomarker for IPF [32]. And another study retrospectively analyzed clinical data from 86 patients with IPF who underwent lung biopsy and found that elevated CRP concentrations at the time of diagnosis of IPF were significantly associated with poor survival [15]. In addition, since acute exacerbation of IPF is life-threatening, Sakamoto et al. performed a logistic regression analysis of information from 103 cases of acute exacerbation of IPF and found that serum CRP was significantly associated with 3-month mortality [17]. And CRP may be a possible biomarker for predicting mortality in patients with acute exacerbations of IPF [16]. In addition, pirfenidone used for the treatment of IPF may reduce CRP by antagonizing NOD-like receptor protein 3 (NLRP3) [33–35]. These suggested that CRP levels may contribute to the pathogenesis and development of IPF. Although all of these studies found an important marker role for circulating CRP levels in the prognosis of IPF, no study clarified the causal effect of elevated CRP on IPF. And these observational studies are susceptible to potential confounding factors or reverse causality. Interestingly, Si et al. evaluated the association of CRP with hundreds of health outcomes using MR analysis and found a negative association between CRP and IPF risk (OR = 0.28, 95% CI 0.15–0.54) [18]. And our study came to a different conclusion, which seems more realistic.

Previous studies have identified environmental pollutants, dust, inflammatory responses, and oxidative stress as potential causes of IPF [36, 37]. In recent years, CRP has been recognized as a systemic marker of chronic inflammation and is an independent risk factor for IPF in many observational studies. Thus, a persistent elevation of circulating CRP levels may represent a state of inflammation, which may increase the risk of IPF. Investigators have found that NLRP3 inflammasome is critical in developing IPF [3]. Pirfenidone, a therapeutic agent for IPF, acts as an antagonist of NLRP3 activation, suggesting that inflammasome may be a potential therapeutic drug target [34]. And a meta-analysis of GWAS indicated that NLRP3 predicted circulating CRP levels [35]. In addition, CRP can stimulate macrophages to produce IL-1 and TNF at sites of inflammation [38], which regulates fibroblast activation, angiogenesis, and extracellular matrix deposition to promote scar tissue formation [39]. Moreover, CRP exhibits a role in promoting organ fibrosis in different organs. You et al. found that CRP may promote renal fibrosis through a TGF-β/Smad3-dependent mechanism [40], while Zhang et al. found that CRP could activate the TGF-β/Smad and NF-kB signaling pathways under high Ang II conditions to promote cardiac fibrosis [41]. Also, the TGF-β/Smad3 pathway plays an important role in pulmonary fibrosis, and inhibition of TGF-β/Smad3 activation can reduce the extent of pulmonary fibrosis [42–44]. Thus, high levels of circulating CRP may increase the risk of IPF by affecting pathways associated with pulmonary fibrosis. These studies provide a possible explanation for the causal effect of circulating CRP levels on the risk of IPF.

Our study has several advantages. Firstly, the present study is the first MR study to assess that elevated circulating CRP levels could increase the risk of IPF and that this association has a causal effect. Secondly, this MR study is based on two large samples of GWAS data from European populations, which provides us with sufficient power to estimate the causal relationship. Thirdly, the MR analysis reveals a long-term effect of genetically determined circulating CRP levels on IPF risk, which is unlikely to be influenced by confounders.

Also, there are some limitations to the study. Firstly, our findings are mainly based on participants of European ancestry and may not apply to populations of other races. Secondly, although we did not find the presence of horizontal pleiotropy, there may be residual bias because the exact function of most of these SNPs is unknown. Thirdly, because our study utilized GWAS summary data and not individual-level data, we were unable to stratify our analysis by other factors such as age and gender. In addition, the circulating CRP levels we studied are genetically controlled, so our results reflected the effect of long-term circulating CRP levels on IPF rather than a short-term response to inflammation.

## Conclusions

Overall, our study indicated that elevated circulating CRP levels could increase the risk of developing IPF. This result probably provides new insight into the understanding of the pathogenesis of IPF. However, further pathological and biochemical studies are needed to investigate further the profound relationship of increased risk of IPF by elevated circulating CRP levels.

## Supplementary Information

**Additional file 1. **The 57 SNPs related to circulating CRP levels at a genome-wide significant level.

**Additional file 2. **The 56 SNPs in IPF GWAS.

**Additional file 3. **IVs for circulating CRP levels.

## Data Availability

All data generated or analysed during this study are included in this published article [and its supplementary information files.

## Abbreviations

IPF: Idiopathic pulmonary fibrosis
CRP: C-reactive protein
MR: Mendelian randomization
GWAS: Genome-wide association studies
IVW: Inverse variance weighted
IV: Instrumental variable
SNP: Single nucleotide polymorphism
NLRP3: NOD-like receptor protein 3
